# Diffuse pattern, orbital invasion, perineural invasion and Ki-67 are associated with nodal metastasis in patients with eyelid sebaceous carcinoma

**DOI:** 10.1136/bjophthalmol-2021-320547

**Published:** 2022-01-21

**Authors:** Xiang Gu, Minyue Xie, Yingxiu Luo, Xin Song, Shiqiong Xu, Xianqun Fan

**Affiliations:** 1 Department of Ophthalmology, Ninth People's Hospital, Shanghai JiaoTong University School of Medicine, Shanghai Key Laboratory of Orbital Diseases and Ocular Oncology, Shanghai, China; 2 Shanghai Key Laboratory of Orbital Diseases and Ocular Oncology, Shanghai, China

**Keywords:** epidemiology, eye lids

## Abstract

**Background:**

Metastasis dominates the prognosis of eyelid sebaceous carcinoma (SC). This study aimed to explore risk factors for nodal metastasis and develop a nomogram to predict nodal metastasis in patients with eyelid SC.

**Methods:**

A retrospective case–control study was performed, comprising 320 patients with eyelid SC. Cox analyses were employed to investigate predictors of metastasis-free survival (MFS), and a nomogram was established and validated by the bootstrap method.

**Results:**

Forty patients (12.5%) developed nodal metastasis during a median follow-up of 48.0 months, and the median period between the initial treatment and first nodal metastasis was 18.5 months (range 6.0–80.0 months). The 1-year, 3-year and 5-year nodal metastasis rates were 5.5%, 12.5% and 15.4%, respectively. Diffuse pattern (HR: 4.34, 95% CI 1.75 to 10.76, p=0.002), orbital invasion at presentation (HR: 3.22, 95% CI 1.42 to 7.33, p=0.005), perineural invasion (HR: 3.24, 95% CI 1.11 to 9.49, p=0.032) and high Ki-67 percentage (HR: 1.03, 95% CI 1.01 to 1.05, p<0.001) were identified as independent risk factors for nodal metastasis. A nomogram that integrated these four factors had a C-index of 0.785, demonstrating a strong power in predicting nodal metastasis of eyelid SC.

**Conclusions:**

We identified risk factors for nodal metastasis and developed a nomogram to provide individualised estimates of nodal metastasis for eyelid SC patients and guide postoperative management. This nomogram contained clinicopathological factors besides the T category of the TNM staging system and suggesting great clinical value.

## Introduction

Sebaceous carcinoma (SC) is a rare eyelid malignancy but is relatively prevalent in Chinese patients. Within all eyelid malignancies, SC accounts for approximately 32.7%–41.6%.^1 2^ It exhibits locally invasive behaviour and performs regional lymph node and distant organ metastasis, leading to a 1.6%–31.0% disease-specific mortality.^3–9^

The predictors for nodal metastasis of eyelid SC are multifactorial and include a prolonged diagnostic delay,^10 11^ involvement of both the upper and lower eyelids,^12^ large tumour size,^6 12 13^ multicentric origin,^12^ diffuse pattern,^11 12^ perivascular invasion,^10^ a non-lobular pattern,^12^ orbital involvement^10^ and an advanced T category.^6–8 12 14 15^ Therefore, it is necessary to identify critical risk factors and develop individualised prediction models, which may contribute to risk stratification and individualised management.

Previous studies have demonstrated that the T category of tumour, node, metastasis (TNM) staging system is often used to predict metastasis of eyelid SC based on the significant correlations between the T category and metastasis.^6–8 14^ However, T category is mainly determined by tumour size, and some other factors, including medical history, growth pattern and pathological features, such as multicentric origin, pagetoid spread, perineural or perivascular invasion, Ki-67 and histology differentiation, have not been included. As a result, it is essential to establish a risk scoring system to take all potential risk factors into consideration.

This retrospective case–control study aimed to explore risk factors for postoperative nodal metastasis of SC and establish a nomogram risk scoring system to predict nodal metastasis in patients with eyelid SC.

## Methods

### Patients

A retrospective, single-centre and case–control study of eyelid SC patients in Ninth People’s Hospital, Shanghai Jiao Tong University School of Medicine was conducted to explore the predictors of nodal metastasis of eyelid SC.

The inclusion criteria were patients who were diagnosed with eyelid SC in Ninth People’s Hospital from January 2005 to December 2020, and the diagnoses were confirmed by pathological examination. The exclusion criteria were as follows: (1) patients with nodal metastasis at presentation; (2) incomplete data collection; (3) less than 6 months of follow-up; and (4) prior periocular irradiation. Of the 390 patients who met the inclusion criteria, 70 patients were excluded, among whom 15 lost follow-ups in the study, 9 had nodal metastasis at presentation, 12 had prior periocular irradiation, 14 had insufficient data (10 due to lack of prior clinicopathological details elsewhere) and 20 were followed up for less than 6 months, leaving a final sample comprising 320 patients. The details of recruitment are in online supplemental figure S1. Informed consent to use their data for research was obtained from all patients.

10.1136/bjophthalmol-2021-320547.supp1 Supplementary data

**Figure 1:**
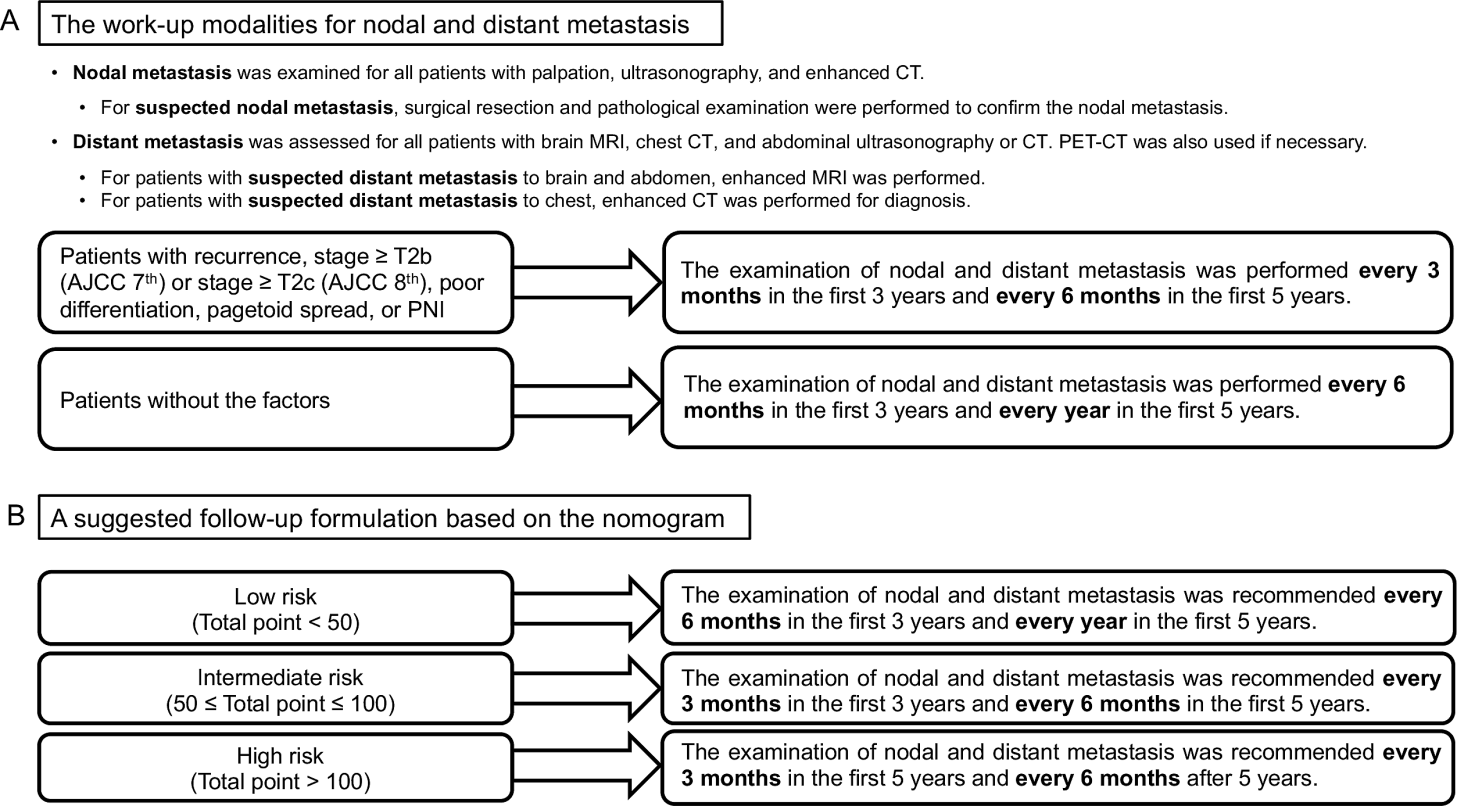
(A) The work-up modalities for nodal and distant metastasis. (B) A suggested follow-up formulation based on the nomogram. AJCC, American joint Committee on Cancer; PET-CT, positron emission tomography/CT.

### Data collection

The data obtained comprised clinical characteristics, pathological features, treatments and outcomes after the follow-up. The clinical and pathological features consisted of age, sex, laterality, prior periocular irradiation, diabetes, diuretic use, hepatitis B virus (HBV) infection, diagnostic delay (period between the appearance of symptoms and the diagnosis), tumour location, initial referral diagnoses, tumour presentation pattern, largest tumour basal diameter, orbital invasion at presentation, pagetoid intraepithelial neoplasia, multicentric origin, perineural invasion (PNI), muscle infiltration, Ki-67 percentage and histological differentiation. According to tumour presentation, eyelid SC was divided into two patterns. A nodular pattern is more common, presenting a solid, firm and distinct nodule in the eyelid, while a diffuse pattern presents a diffuse unilateral thickening of the eyelid, lacks a well-defined margin and exhibits an inflammatory appearance.^16^ Of note, the degree of differentiation was subdivided according to a previous report. Well differentiation manifested as lobules with sebaceous differentiation, moderately differentiation comprised anaplastic cells with highly differentiated sebaceous cells and poorly differentiation manifested as tumours filled with pleomorphic nuclei, prominent nucleoli and amphophilic-positive cytoplasm.^17^ The eighth edition of the American Joint Committee on Cancer (AJCC) staging system was used to stratify the patients. The surgical approaches included frozen margin control, wide local excision and orbital exenteration. Frozen margin control means all the excised specimens underwent frozen section studies of the margins. If any of the margins were positive, repeated excisions were performed until all the margins were negative, which guarantees patients to obtain intraoperative margin clearance on frozen sections.^18^ Wide local excision with 5 mm margins of normal-appearing tissue confirmed all surgical margins pathologically with permanent parafﬁn section analysis 1 week after the excision. The status of surgical margins recorded means whether the tumour was detected on the permanent section after the initial surgery. If positive, a further excision was performed. Moreover, orbital exenteration was performed for patients with extensive involvement of orbital or periorbital anatomy. The outcome measures were time between the initial treatment and nodal metastasis or death. The confirmation of nodal or distant metastasis depended on pathology or imaging. The work-up modalities for nodal and distant metastasis were in figure 1A.

### Statistical analysis

The analyses were implemented employing SPSS software (V.26.0, IBM) and the corrplot package (V.0.84), rms package (V.3.1), pROC package (V.1.17.0.1) and ggplot2 package (V.3.2.1) in R version 3.6.1 (The R Foundation). Categorical variables are described as the frequency (percentage), and continuous variables with a skewed distribution are described as the median (range). The χ^2^ test (or Fisher’s exact test, if applicable) and the non-parametric Mann-Whitney U test were employed to compare categorical variables and continuous variables with skewed distributions, respectively. Survival and metastasis rates were calculated based on Kaplan-Meier analysis and were compared by the log-rank test. A value of p<0.05 was considered statistically significant.

Univariate Cox proportional hazards regression was used to identify the potential predictors of nodal metastasis in patients with eyelid SC from demographic and clinicopathological indicators. The significant factors (p<0.05) that were subjected to correlation analysis were then inputted into the stepwise multivariate Cox proportional hazards regression analysis, and the significant variables (p<0.05) in the final multivariate Cox regression model were considered independent predictors. The HRs with 95% CIs in the Cox regression analysis were recorded.

A nomogram was then established according to the final multivariate Cox regression model, and five criteria were employed to evaluate the prediction performance (discrimination and calibration) of the nomogram.^19^ First, the patients were grouped according to their predicted risk score, and differences in nodal metastasis across groups were compared by Kaplan-Meier analysis and the log-rank test. Second, the concordance index (C-index) was calculated to assess the discrimination ability of the model, where a larger C-index means a greater discrimination ability. Third, the area under the curve (AUC) of the time-dependent receiver operating characteristic curve was calculated and compared with that of the T category by the DeLong test. Fourth, the net reclassification improvement (NRI) and the integrated discrimination improvement (IDI) were used to compare the nomogram with the T category. All four criteria represent the discrimination ability of the model, the similarity between predicted and observed metastasis-free outcomes and the predicted risk score. Finally, calibration curves of the nomogram for 1-year, 3-year and 5-year metastasis-free survival (MFS) were generated to evaluate the agreement between the predicted and observed outcomes. Relatively unbiased estimates of the performance were achieved by the bootstrapping method (1000 repetitions). All analyses were two sided, and p value <0.05 was statistically significant.

## Results

### Demographic information, clinicopathological characteristics and treatment data

We enrolled 320 patients in this study, in which the median age at diagnosis was 63.0 years, ranging from 27.0 to 94.0 years. There were 136 (42.5%) male patients and 184 (57.5%) female patients. Thirty-five (10.9%) patients presented positive surgical margins on the permanent sections after the initial treatment, among whom 34 (10.6%) had experienced wide local excision and 1 (0.3%) had experienced frozen margin control. These patients were surgically resected for the second time and achieved negative margins on the permanent sections. Eight (2.5%) of these 35 (10.9%) patients experienced subsequent local recurrence and positive surgical margins presented no significant correlation to local recurrence (p=0.481). After a median follow-up time of 48.0 months (range 6.0–178.0 months), 40 patients (12.5%) developed nodal metastasis and 57 patients (17.8%) experienced local recurrence. The locations of the initial metastases included the preauricular lymph nodes (n=28 (8.8%)), submandibular lymph nodes (n=17 (5.3%)), cervical lymph nodes (n=8 (2.5%)) and postauricular lymph nodes (n=1 (0.3%)). Among these patients who developed nodal metastasis, 15 (4.7%) presented with distant metastasis to lung. The median period from the initial treatment to first nodal metastasis was 18.5 months (range 6.0–80.0 months) and from the initial treatment to first distant metastasis, it was 31.0 months (range 12.0–80.0 months). According to Kaplan-Meier survival estimates, the 1-year, 3-year and 5-year nodal metastasis rates were 5.5%, 12.5% and 15.4%, respectively. The demographic information, clinical features, initial treatments and pathological characteristics of patients with and without nodal metastasis are compared in table 1. The two groups exhibited significant differences in medial canthus involvement (p=0.004), tumour presentation pattern (p=0.036), greatest basal diameter (p=0.003), orbital invasion at presentation (p<0.001), PNI (p=0.015), Ki-67 percentage (p<0.001), T category (p=0.002) and initial treatment (p<0.001). After the follow-up period, 283 (88.4%) patients were no evidence of disease, 0 patient was alive with disease and 37 (11.6%) patients died, among whom 16 (5.0%) died of the disease and 21 (6.6%) died of unrelated cause. According to Kaplan-Meier survival estimates, the 5-year and 10-year disease-specific survival rates were 94.2% and 92.1%, respectively.

**Table 1:** The demographic and clinical characteristics of patients with or without nodal metastasis

	Total (n=320)	Metastasis (n=40)	No metastasis (n=280)	P value
Age, median (min, max), years	63.0 (27.0, 94.0)	63.0 (34.0, 85.0)	63.0 (27.0, 94.0)	0.226
Sex, no. (%)				0.305
Male	136 (42.5)	14 (35.0)	122 (43.6)	
Female	184 (57.5)	26 (65.0)	158 (56.4)	
Laterality, no. (%)				0.933
Right	154 (48.1)	19 (47.5)	135 (48.2)	
Left	166 (51.9)	21 (52.5)	145 (51.8)	
Diabetes, no. (%)	26 (8.1)	2 (5.0)	24 (8.6)	0.643
History of diuretic use, no. (%)	31 (9.7)	3 (7.5)	28 (10.0)	0.830
HBV, HBsAg (+), no. (%)	12 (3.8)	0	12 (4.3)	0.374
Diagnostic delay, median (min, max), months	12.0 (0.3, 180.0)	12.0 (0.3, 72.0)	12.0 (0.5, 180.0)	0.628
Location, no. (%)				
Upper lid	190 (59.4)	23 (57.5)	167 (59.6)	0.796
Lower lid	130 (40.6)	17 (42.5)	113 (40.4)	0.796
Both upper lid and lower lid	13 (4.1)	3 (7.5)	10 (3.6)	0.454
Medial canthus	24 (7.5)	8 (20.0)	16 (5.7)	0.004*
Initial diagnosis, no. (%)				0.313
Sebaceous carcinoma	219 (68.4)	29 (72.5)	190 (67.9)	
Squamous cell carcinoma	24 (7.5)	2 (5.0)	22 (7.9)	
Basal cell carcinoma	13 (4.1)	1 (2.5)	12 (4.3)	
Merkel cell carcinoma	1 (0.3)	0	1 (0.4)	
Chalazion	19 (5.9)	2 (5.0)	17 (6.1)	
Blepharitis	33 (10.3)	2 (5.0)	31 (11.1)	
Dermoid	11 (3.4)	4 (10.0)	7 (2.5)	
Tumour presentation pattern				0.036*
Nodule	300 (93.8)	34 (85.0)	266 (95.0)	
Diffuse	20 (6.3)	6 (15.0)	14 (5.0)	
Greatest basal diameter, median (min, max), mm	10.0 (2.0, 50.0)	13.5 (3.0, 40.0)	10.0 (2.0, 50.0)	0.003*
Orbital invasion at presentation, no. (%)	20 (6.3)	8 (20.0)	12 (4.3)	<0.001*
Pagetoid spread, no. (%)	105 (32.8)	14 (35.0)	91 (32.5)	0.948
Multicentric origin, no. (%)	36 (11.3)	7 (17.5)	29 (10.4)	0.285
PNI, no. (%)	9 (2.8)	4 (10.0)	5 (1.7)	0.015*
Perivascular invasion, no. (%)	5 (1.6)	1 (2.5)	4 (1.8)	0.489
Muscle infiltration, no. (%)	50 (15.6)	10 (25.0)	40 (14.3)	0.081
Ki-67 percentage, median (min, max)	35.0 (0, 90.0)	50.0 (0, 80.0)	30.0 (0, 90.0)	<0.001*
Histological differentiation, no. (%)				0.063
Well	47 (14.7)	4 (10.0)	43 (14.9)	
Moderate	230 (71.9)	26 (65.0)	204 (73.3)	
Poor	43 (13.4)	10 (25.0)	33 (11.8)	
T category, no. (%)				0.002*
T1	189 (59.1)	16 (40.0)	173 (61.8)	
T2	84 (26.3)	12 (30.0)	72 (25.7)	
T3	27 (8.4)	4 (10.0)	23 (8.2)	
T4	20 (6.3)	8 (20.0)	12 (4.3)	
With positive surgical margin, no. (%)	35 (10.9)	6 (15.0)	29 (10.4)	0.542
Local recurrence	57 (17.8)	9 (22.5)	48 (17.1)	0.407
Initial treatment, no. (%)				<0.001*
Frozen margin control	159 (49.7)	7 (17.5)	152 (54.3)	
Wide local excision	149 (46.6)	27 (67.5)	122 (43.6)	
Orbital exenteration	12 (3.8)	6 (15.0)	6 (2.1)	

*Statistically signiﬁcant.

HBsAg, hepatitis B surface antigen; HBV, hepatitis B virus; PNI, perineural invasion; T, Tumour category according to the eighth edition of the American Joint Committee on Cancer staging system.

### Cox regression analysis for predictors of nodal metastasis

To investigate the independent risk factors for nodal metastasis of eyelid SC, univariable and multivariable Cox regression analyses were implemented and are summarised in table 2. The correlation analysis suggested that the T category was strongly correlated with the greatest basal diameter (R=0.80, p<0.001) and orbital invasion (R=0.70, p<0.001), and the greatest basal diameter and orbital invasion presented with more detailed clinical information; therefore, the T category was not included in the analysis (online supplemental figure S2). In the univariate analysis, medial canthus involvement (p=0.003), diffuse pattern (p=0.001), greatest basal diameter (p=0.008), orbital invasion at presentation (p<0.001), PNI (p<0.001), high Ki-67 percentage (p<0.001) and orbital exenteration (orbital exenteration versus frozen margin control, p<0.001, orbital exenteration versus wide local excision, p<0.001) were identified as potential risk factors for nodal metastasis of eyelid SC. However, the differences in initial treatment between orbital exenteration and frozen margin control or wide local excision were due to a tight association between orbital exenteration and orbital invasion at presentation, and orbital invasion at presentation was already considered a potential risk factor; therefore, initial treatment was excluded in the following analysis. The factors medial canthus involvement, diffuse pattern, greatest basal diameter, orbital invasion at presentation, PNI and Ki-67 percentage were included in the subsequent multivariable analysis, and the final multivariable model suggested that diffuse pattern (HR: 4.34, 95% CI 1.75 to 10.76, p=0.002), orbital invasion at presentation (HR: 3.22, 95% CI 1.42 to 7.33, p=0.005), PNI (HR: 3.24, 95% CI 1.11 to 9.49, p=0.032) and high Ki-67 percentage (HR: 1.03, 95% CI 1.01 to 1.05, p<0.001) were independent predictors of nodal metastasis.

10.1136/bjophthalmol-2021-320547.supp2 Supplementary data

**Figure 2:**
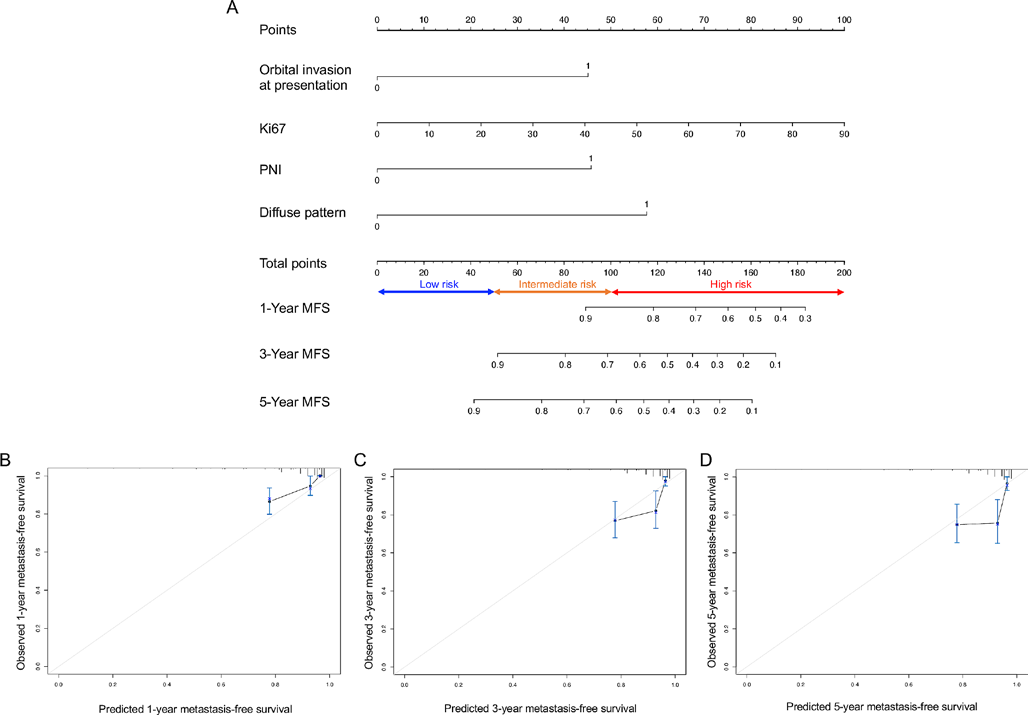
Nomogram for metastasis-free survival (MFS). (A) Nomogram to predict the probability of MFS at 1, 3 and 5 years. Instructions for the nomogram: draw a vertical line for each variable to the ‘points’ line to attain the score of each factor and sum the scores. Then, put the total score on the ‘total points’ line and draw a vertical line to the 1-year, 3-year and 5-year survival probability lines to determine the probability of 1-year, 3-year and 5-year MFS. The total point of the nomogram is used to classify patients into low-risk (less than 50), intermediate-risk (50–100) and high-risk groups (more than 100). (B–D) Calibration plots for metastasis-free survival probability at 1 year (B), 3 years (C) and 5 years (D) for MFS probability. The x-axis indicates the nomogram-predicted survival probability, and the y-axis indicates the observed survival probability. The vertical lines indicate the 95% conﬁdence intervals of the estimates. The grey line represents that the predicted probabilities are identical to the observed probabilities. Black dot: predicted probabilities according to the nomogram; blue cross: bootstrap-corrected estimates. B=1000 repetitions for bootstrapping. MFS, metastasis-free survival; PNI, perineural invasion.

**Table 2:** Univariable and multivariable COX proportional hazards regression analysis for the predictors of nodal metastasis

	Univariable		Multivariable	
	HR (95% CI)	P value	HR (95% CI)	P value
Age, years	0.99 (0.97 to 1.02)	0.454		
Sex (female vs male)	1.40 (0.73 to 2.68)	0.309		
Laterality (left vs right)	1.16 (0.62 to 2.16)	0.648		
Diabetes	0.61 (0.15 to 2.55)	0.502		
History of diuretic use	0.74 (0.23 to 2.39)	0.613		
HBV, HBsAg (+)	0.05 (0 to 29.44)	0.351		
Diagnostic delay, months	1.00 (0.99 to 1.01)	0.550		
Upper lid	0.97 (0.52 to 1.82)	0.926		
Lower lid	1.08 (0.58 to 2.02)	0.814		
Both upper lid and lower lid	2.21 (0.68 to 7.18)	0.186		
Medial canthus	3.26 (1.50 to 7.08)	0.003*	1.09 (0.35 to 3.44)	0.878
Initial diagnosis (others vs sebaceous carcinoma)	0.76 (0.38 to 1.53)	0.442		
Tumour presentation pattern (diffuse vs nodule)	4.16 (1.73 to 10.00)	0.001*	4.34 (1.75 to 10.76)	0.002*
Greatest basal diameter, mm	1.05 (1.01 to 1.08)	0.008*	1.02 (0.99 to 1.06)	0.220
Orbital invasion at presentation	3.98 (1.83 to 8.65)	<0.001*	3.22 (1.42 to 7.33)	0.005*
Pagetoid spread	1.02 (0.52 to 2.01)	0.953		
Multicentric origin	1.88 (0.83 to 4.25)	0.130		
PNI	6.51 (2.30 to 18.42)	<0.001*	3.24 (1.11 to 9.49)	0.032*
Perivascular invasion	2.27 (0.31 to 16.57)	0.419		
Muscle infiltration	1.76 (0.86 to 3.61)	0.121		
Ki-67 percentage	1.04 (1.02 to 1.05)	<0.001*	1.03 (1.01 to 1.05)	<0.001*
Histological differentiation				
Moderate versus well	1.28 (0.45 to 3.66)	0.650		
Poor versus well	2.67 (0.84 to 8.53)	0.097		
With positive surgical margin	1.01 (0.42 to 2.41)	0.991		
Local recurrence	1.23 (0.58 to 2.58)	0.590		
Initial treatment				
Wide local excision versus frozen margin control	2.05 (0.87 to 4.87)	0.103		
Orbital exenteration versus frozen margin control	10.29 (3.44 to 30.75)	<0.001*		
Wide local excision versus orbital exenteration	0.20 (0.08 to 0.49)	<0.001*		

*Statistically signiﬁcant.

HBV, hepatitis B virus; PNI, perineural invasion.

### A nomogram to predict nodal metastasis

**Figure 3:**
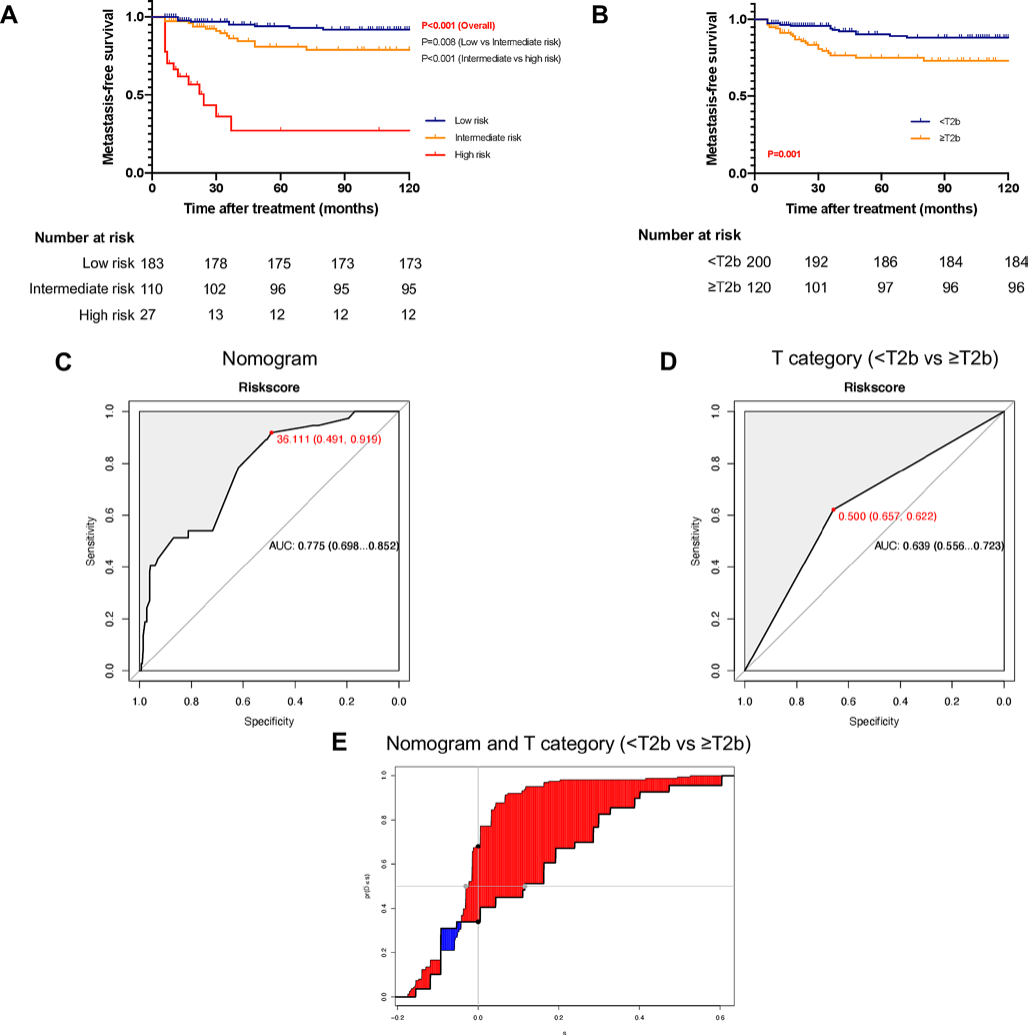
The discrimination accuracy of the nomogram and T category (<T2b vs ≥T2b). (A) Kaplan-Meier curves of metastasis-free survival for the low-risk, intermediate-risk and high-risk groups stratiﬁed by the nomogram. (B) Kaplan-Meier curves of metastasis-free survival based on the T category (<T2b vs ≥T2b). (C) Receiver operating characteristic (ROC) curve for the nomogram predicting 5-year metastasis-free survival; the area under the curve (AUC) of the time-dependent ROC curve was 0.775. (D) ROC curves according to the T category (<T2b vs ≥T2b); the AUC of the time-independent ROC curve was 0.639. (E) The integrated discrimination improvement between the nomogram and the T category (<T2b vs ≥T2b) was 0.143 (0.043, 0.298).

A predictive nomogram that incorporated all independent predictors for nodal metastasis was constructed, and the final risk score was calculated by summing the score of each item using the nomogram illustrated in figure 2A. The C-index for this model was 0.785, indicating a high consistency between the predicted and observed probabilities of nodal metastasis. The calibration curves for 1-year, 3-year and 5-year MFS also demonstrated fair agreement between the predicted and actual observations (figure 2B–D). The Cox regression model of the risk score was used to classify patients into low-risk (total point less than 50), intermediate-risk (total point 50–100) and high-risk groups (total point more than 100), and the MFS times were significantly different among the groups (figure 3A; log-rank p<0.001 (overall); p=0.008 (low vs intermediate risk); p<0.001 (intermediate vs high risk)).

### The nomogram compared with the T category

The discrimination accuracy between the nomogram and the T category was demonstrated as follows. The AUC of the nomogram for predicting 5-year MFS was 0.775 (figure 3C), higher than that of the T category (T1 vs T2 vs T3 vs T4, 0.650, p=0.025, online supplemental figure S3C; <T2b vs≥T2b, 0.639, p=0.014, figure 3D;<T2c vs ≥T2c, 0.628, p=0.006, online supplemental figure S3D). The C-index of this nomogram was 0.785, which was higher to the C-indexes of the T category (T1 vs T2 vs T3 vs T4, 0.635, p<0.001; <T2b vs ≥T2b, 0.631, p=0.001; <T2c vs ≥T2c, 0.604, p=0.001). In addition, the NRI between the nomogram and the T category (T1 vs T2 vs T3 vs T4, <T2b vs ≥T2b and <T2c vs ≥T2c) was 0.278 (−0.030, 0.527), 0.341 (−0.064, 0.554) and 0.343 (0.068, 0.575), respectively. The IDI between the nomogram and the T category (T1 vs T2 vs T3 vs T4, <T2b vs ≥T2b and <T2c vs ≥T2c) was 0.125 (0.042, 0.264) (online supplemental figure S3E), 0.143 (0.043, 0.298) (figure 3E) and 0.155 (0.062, 0.320) (online supplemental figure S3F), respectively. Furthermore, Kaplan-Meier analysis was employed based on the T category (T1 vs T2 vs T3 vs T4, log-rank p<0.001 (overall), p=0.096 (T1 vs T2); p=0.951 (T2 vs T3); p=0.077 (T3 vs T4), online supplemental figure S3A;<T2b vs ≥T2b, log-rank p=0.001, figure 3B;<T2c vs ≥T2c, log-rank p=0.003, online supplemental figure S3B). These results confirmed the strong power of this proposed nomogram in predicting nodal metastasis.

10.1136/bjophthalmol-2021-320547.supp3 Supplementary data

## Discussion

The study was the largest single-centre retrospective series of eyelid SC and provided detailed information on nodal metastasis. The nodal metastasis rate in the study was 12.5%, which was in accordance with those from the previous multicentre cohort study in mainland China (13.1%) and the study by Lam *et al* reported in Hong Kong (13.6%), higher than those reported by Takahashi *et al* in Japan (5.9%) and Shields *et al* in America (8.0%) but lower than those reported by Sa *et al* in America (21.0%) and Hsia *et al* in Taiwan (22.0%). These findings indicate that Chinese patients with eyelid SC have comparable probabilities of nodal metastasis to patients from other countries or regions, and the existing discrepancies may have resulted from analyses of different races, different follow-up periods and multiple management methods.

In this study, we established a novel nomogram that incorporates four independent risk factors for MFS to predict nodal metastasis: diffuse pattern, orbital invasion at presentation, PNI and high Ki-67 percentage. The diffuse pattern frequently induces blepharoconjunctivitis, which is the major cause of delayed diagnosis and makes complete resection of lesions difficult.^4 20^ Therefore, this pattern is associated with local recurrence and nodal metastasis, as previously reported and consistent with our results.^12^ Orbital invasion is the principal determinant of the T category and has been demonstrated to be associated with a poor prognosis and nodal metastasis.^10 14 21^ PNI refers to the histological infiltration of tumour cells into the sheaths of peripheral nerves adjoining the primary lesion.^22^ Although the clinical practice guidelines of SC proposed that the influence of PNI on prognosis is uncertain owing to its low incidence in SC,^23^ emerging evidence has suggested that supportive cells inside peripheral nerves cooperate with tumour cells and directly promote tumour invasion and dissemination along nerves.^24–26^ In addition, PNI is associated with aggressive tumour behaviours and adverse clinical outcomes in basal cell carcinoma and squamous cell carcinoma.^27 28^ Ki-67, a general marker for tumour cell proliferation, is also correlated with nodal metastasis in multiple types of cancer, such as nasopharyngeal carcinoma, laryngeal carcinoma, prostate cancer and breast cancer.^29–32^ For eyelid carcinoma, Ki-67 is a sensitive indicator for a malignant tumour grade and is significantly correlated with infiltrative growth in SC, indicating its potential ability to predict nodal metastasis of eyelid SC.^33 34^

Of note, initial treatment demonstrated a significant difference in the patients with and without nodal metastasis, frozen margin control accounted for 17.5%, while wide local excision accounted for 67.5% in patients with nodal metastasis; however, in the univariable Cox proportional hazards regression analysis, initial treatment (wide local excision vs frozen margin control) presented no significant difference (p=0.103). The main reason was that frozen margin control had been performed in our centre since 2012, resulting in a shorter follow-up period of patients with frozen margin control than wide local excision.

Nomogram is a visual tool that employs statistical models to provide personalised risk estimates and improve management-related decisions in various carcinomas.^35^ A robust nomogram was established in our study to predict the development of nodal metastasis of eyelid SC and therefore contributes to the postoperative hierarchical management and selection of candidates for trials designed to evaluate preventive interventions. A suggested follow-up formulation based on the risk stratification of this nomogram was in figure 1B. In addition, our nomogram proposed the importance of pathological factors in predicting nodal metastasis, which may have implications for future AJCC classifications.

This study has some limitations. Although the nomogram was internally validated by bootstrapping, it was not externally validated using an independent data set from another institution. Moreover, all the patients recruited for this study were from China. As a result, the generalisation of this nomogram to other institutions and other populations remains unclear. In addition, the patients who were excluded from our study due to their inability to reach or their age and/or health condition may have produced selection bias. Furthermore, eight (2.5%) patients included in our study that had previously treated elsewhere, five (1.6%) of whom came to our centre due to their awareness of recurrence and three (0.9%) of whom due to positive surgical margin. The inclusion could make selection bias. The relatively small number of outcome events in this study may have caused large CIs for estimates in the calibration plot. Therefore, a broader population and a larger sample size of patients with eyelid SC are necessary to further evaluate the discrimination and calibration abilities of the model.

In conclusion, we identified risk factors for nodal metastasis and developed and validated a novel predictive nomogram using various methods to offer accurate individualised estimates for the nodal metastasis of eyelid SC. The proposed nomogram comprised clinicopathological factors besides the T category of TNM staging system and expressed great potential in clinical application.

## Data Availability

All data relevant to the study are included in the article or uploaded as supplementary information. All datasets generated for this study are included in the article.
