# Supplementary figures and images for: Diffuse pattern, orbital invasion, perineural invasion and Ki-67 are associated with nodal metastasis in patients with eyelid sebaceous carcinoma
Source: Br J Ophthalmol. 2022 Jan 21;107(6):756–62. doi: 10.1136/bjophthalmol-2021-320547 (PMC10314074; doi:10.1136/bjophthalmol-2021-320547)

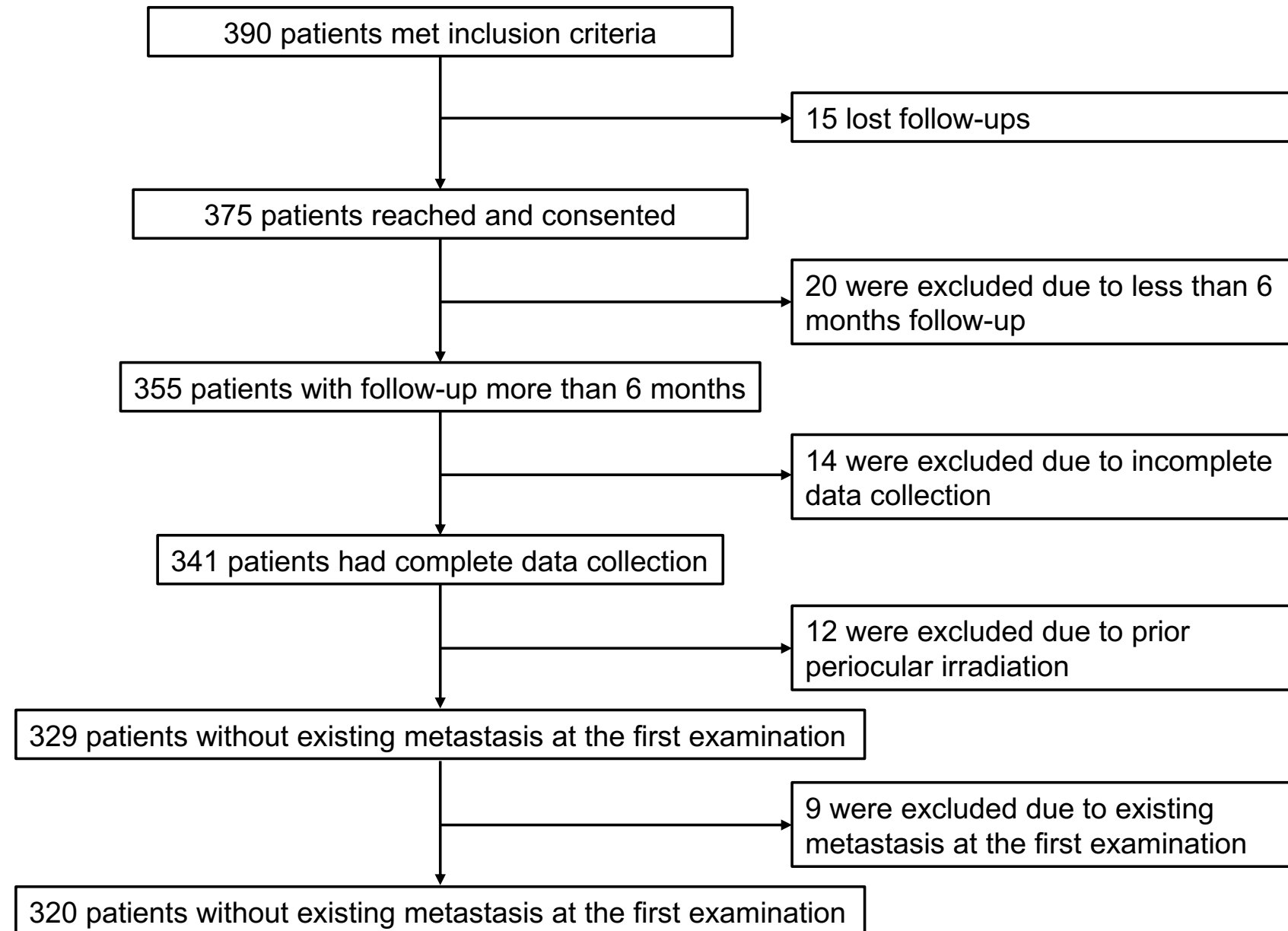

Supplement: Supplementary data [file bjophthalmol-2021-320547supp001.pdf]

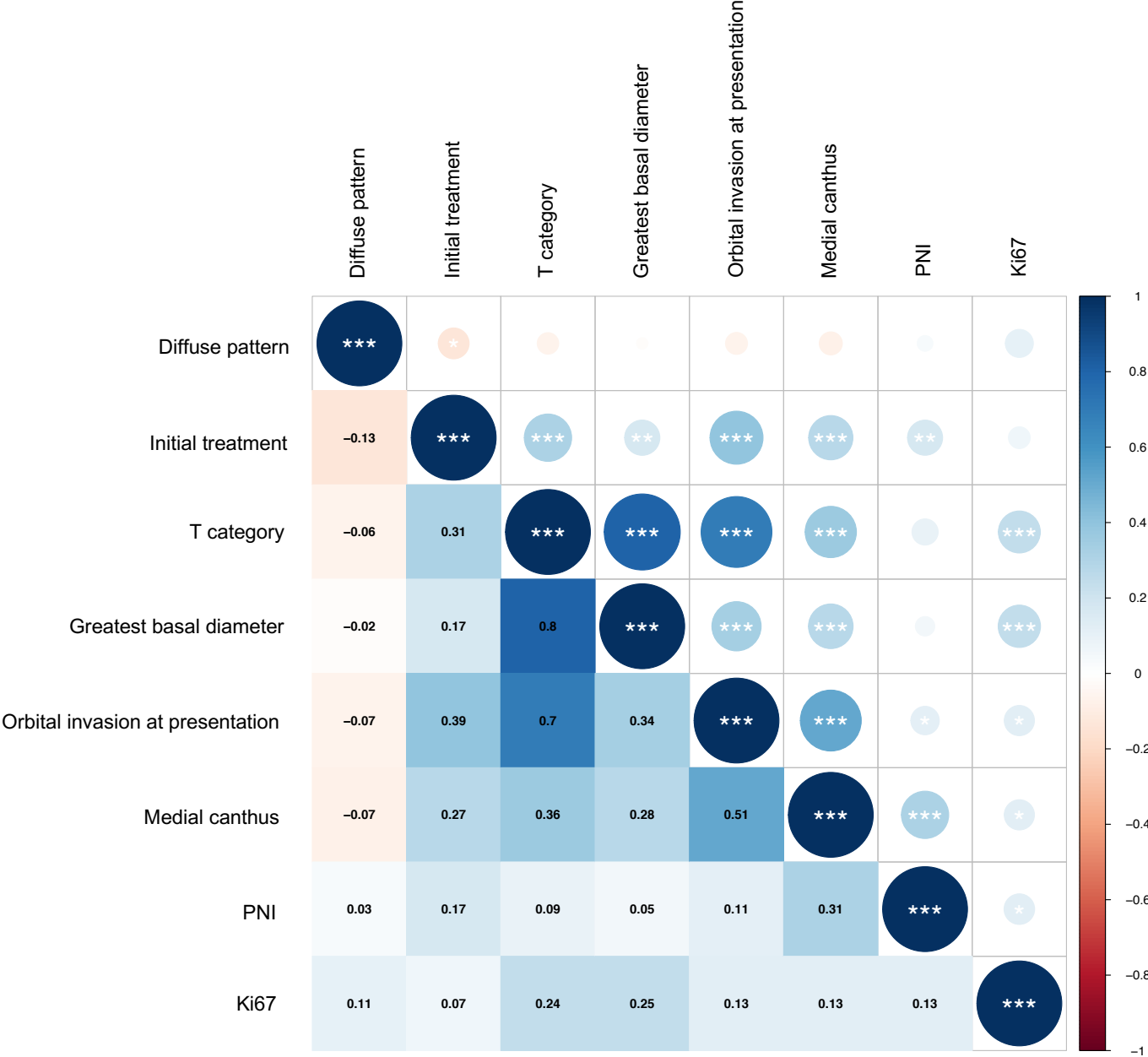

Supplement: Supplementary data [file bjophthalmol-2021-320547supp002.pdf]

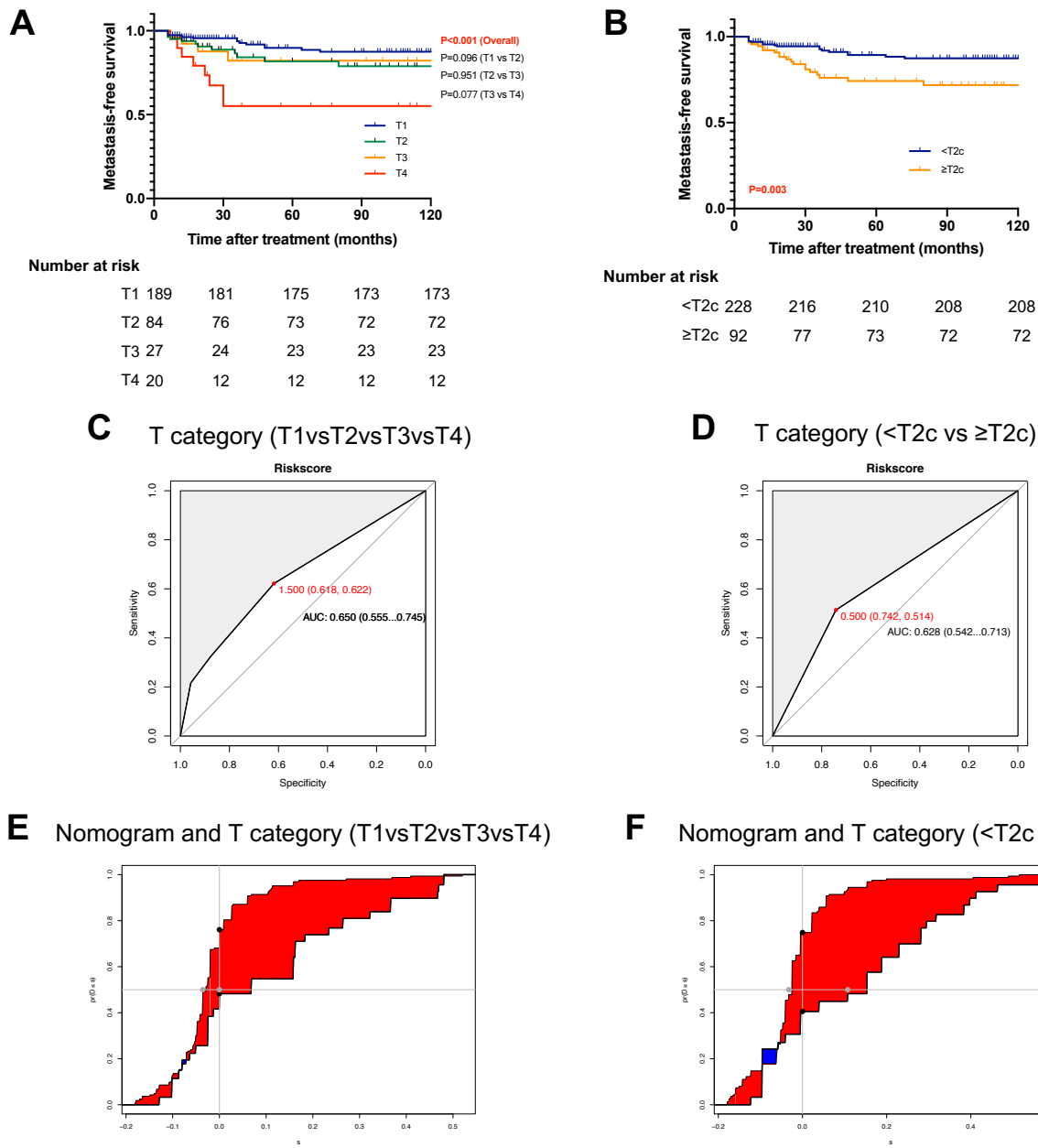

Supplement: Supplementary data [file bjophthalmol-2021-320547supp003.pdf]
